# Investigation using whole genome sequencing of a prolonged restaurant outbreak of *Salmonella* Typhimurium linked to the building drainage system, England, February 2015 to March 2016

**DOI:** 10.2807/1560-7917.ES.2017.22.49.17-00037

**Published:** 2017-12-07

**Authors:** John Mair-Jenkins, Roberta Borges-Stewart, Caroline Harbour, Judith Cox-Rogers, Tim Dallman, Philip Ashton, Robert Johnston, Deborah Modha, Philip Monk, Richard Puleston

**Affiliations:** 1 Field Epidemiology Training Programme, Public Health England, United Kingdom; 2 European Programme for Intervention Epidemiology Training (EPIET), European Centre for Disease Prevention and Control (ECDC), Stockholm, Sweden; 3 Field Epidemiology Service, National Infection Service, Public Health England, United Kingdom; 4 East Midlands Health Protection Team, Public Health England, United Kingdom; 5 Environmental Health, Blaby District Council, Blaby, United Kingdom; 6 Gastrointestinal Bacteria Reference Unit, National Infection Service, Public Health England, United Kingdom; 7 Food Water and Environment Laboratory, National Infection Service, Public Health England, United Kingdom; 8 Clinical Microbiology, Leicester Royal Infirmary, University Hospitals of Leicester NHS Trust, United Kingdom; 9 University of Nottingham, School of Medicine, Division of Epidemiology and Public Health, United Kingdom

**Keywords:** Salmonellosis, Gastrointestinal disease, Salmonella, outbreaks, food-borne infections

## Abstract

Following notification of a *Salmonella enterica* serovar Typhimurium gastroenteritis outbreak, we identified 82 cases linked to a restaurant with symptom onset from 12 February 2015 to 8 March 2016. Seventy-two cases had an isolate matching the nationally unique whole genome sequencing profile (single nucleotide polymorphism (SNP) address: 1.1.1.124.395.395). Interviews established exposure to the restaurant and subsequent case–control analysis identified an association with eating carvery buffet food (adjusted odds ratios (AOR): 20.9; 95% confidence interval (CI): 2.2 – ∞). Environmental inspections, food/water testing, and a food trace-back investigation were inconclusive. Repeated cycles of cleaning were undertaken, including hydrogen peroxide fogging, however, transmission continued. After 7 months of investigation, environmental swabbing identified 106 isolates from kitchen surfaces and restaurant drains matching the outbreak profile. We found structural faults with the drainage system and hypothesised that a reservoir of bacteria in drain biofilm and underfloor flooded areas may have sustained this outbreak. Ineffective drain water-traps (U-bends) may have also contributed by allowing transmission of contaminated aerosols into the kitchen environment. These findings suggest that routine swabbing of sink drain points and inspection of drainage systems should be considered in future outbreak scenarios.

## Introduction

It is estimated that over 38,000 community cases of salmonellosis occur annually within the United Kingdom (UK) [1,2]. Salmonellosis often results from consumption of contaminated food or water [3], however, transmission via asymptomatic shedding by food handlers and exposure to contaminated environments where conditions are favourable for pathogen survival have also been implicated [3,4]. Here we report the findings of an investigation of an outbreak of salmonellosis where the environment was pivotal in continued transmission.

### The event

On 7 March 2015, Public Health England (PHE) East Midlands was alerted by the clinical microbiology laboratory of a local hospital to 21 cases of *Salmonella enterica* serovar Typhimurium gastroenteritis, with onset in February 2015. Seven cases in this initial phase of the outbreak required hospitalisation. Following this notification we suspected there was a community outbreak of *S.* Typhimurium; investigations and attempts to control the outbreak followed.

Hypothesis-generating interviews at the outset of the investigation identified that several cases had eaten at the same restaurant during the incubation period for their illness. Descriptive epidemiological analyses including subsequent cases pointed to the restaurant being the likely source. This popular, purpose (newly) built restaurant had opened only 18 months before the outbreak. The restaurant offered a full table-service menu, self-service salad bar and hot self-service carvery buffet serving roasted meats (turkey, beef, gammon and pork at weekends) and vegetables and condiments. Despite interventions to control the initial outbreak, cases continued to emerge followed by a prolonged period of transmission until 2016. The evolution of the investigation into this community outbreak and subsequent control measures is described, with specific reference to the use of whole genome sequencing (WGS) to link isolates and the role of the drains in continued pathogen transmission.

## Methods

### Epidemiological investigations

Case finding used information from existing cases, local primary care practitioners and pre-existing surveillance systems (statutory disease and routine laboratory notifications, including routine WGS) [5]. Active case finding using restaurant booking information was not possible as most customers did not pre-book.

Hypothesis-generating interviews and descriptive analyses informed a case–control study in March 2015 where we defined confirmed cases as UK residents with *S.* Typhimurium infection (with or without gastroenteritis) with an isolate matching the nationally unique WGS outbreak profile (five single nucleotide polymorphisms (SNP) single linkage cluster 1.1.1.124.395.395) [6] with symptom onset or a positive sample taken within 12 days of visiting the restaurant. Possible cases were defined as having gastroenteritis (diarrhoea – three loose stools within 24 hours – or any two of abdominal pain, fever or nausea) within 12 days of visiting the restaurant [7,8].

As part of the outbreak investigations a case–control study was undertaken, to understand the association between consuming restaurant food or drink and illness, with the assumption that a food associated source was the most likely vector. Cases were recruited and were requested to nominate people who had eaten with them as controls (1:1 ratio). No other criteria were set for control selection. After data collection any controls who met case definitions were reassigned as cases. We asked about exposure to food and drink and potential confounding exposures: immunosuppression, recent use of antibiotics or antacids, age and sex. We calculated univariate odds ratios (OR), and adjusted ORs (aOR) using multivariable exact logistic regression. Due to continued case occurrences after the implementation of outbreak control measures (as informed by the findings of the case–control study), further investigations ensued, including increased scrutiny of the environment.

### Clinical microbiological investigations

Stool samples were collected for culture and characterisation following clinical investigations and voluntary sampling of restaurant staff in March 2015 and again following further case occurrences in November 2015. Positive isolates were submitted to the PHE Gastrointestinal Bacteria Reference Unit (GBRU) for serological confirmation, phage typing and multilocus variable-number tandem repeat analysis (MLVA), until April 2015. Extracted DNA from isolates was also sequenced using Nextera library preparation (Illumina, Inc. San Diego, California, United States) on an Illumina HiSeq 2500 machine. High quality Illumina reads were mapped to the *S*. Typhimurium reference genome (GenBank: AE006468) as previously described [9]. Core genome positions that had a high quality SNP (> 90% consensus, minimum depth 10×, genotype quality ≥ 30) in at least one strain were extracted and RaxMLv8.17 (Stamatakis, 2014) used to derive the maximum likelihood phylogeny of the isolates under the general time-reversible (GTR)-CAT model of evolution. Single linkage SNP clustering was performed [9]. FASTQ reads from all sequences in this study can be found at the PHE Pathogens BioProject at the National Center for Biotechnology Information (Accession PRJNA248792). Isolates within the five SNP single linkage cluster 1.1.1.124.395.395 were defined as part of the outbreak.

### Environmental investigations and site visits

Environmental Health Officers (EHOs) carried out a routine inspection at the restaurant and reviewed staff sickness records when the outbreak was first identified. Surface swabs using Dacron tipped swabs and sponge swabs, along with food and water samples were collected using standard techniques [10]. Repeat site visits and inspections continued throughout the outbreak. A food trace-back investigation was conducted to identify the suppliers of meat, eggs, stuffing and gravy to establish if local suppliers were being used or whether larger national/international suppliers had links to other reported cases or outbreaks of *S*. Typhimurium.

In November 2015, following failure to control the outbreak, we considered potential environmental sources that could provide an ongoing intermittent exposure which had not previously been investigated. Repeated cycles of cleaning were undertaken at the restaurant, including deep cleans using hydrogen peroxide fogging, however these still failed to control the incident. Further investigations including a Water Fittings Inspection [11], sewer sampling using a non-validated method similar to a Moore swab [12] and an inspection of the restaurant drainage systems were undertaken. The drains were visually inspected and microbiological sampling of the kitchen floor drains and sink drains was undertaken. Expert advice was sought on the movement of bio-aerosols along drainage systems [13]. Drainage systems were inspected internally using remote cameras, swabbing was conducted and smoke testing was used to establish air flows and the integrity of the drainage. Repeat inspections and environmental swabbing were undertaken after remedial works.

Environmental samples were submitted to PHE Food, Water and Environmental Microbiology Laboratories. These were tested for *Salmonella* sp. using routine detection methods for surface swabs and food [14]. Testing methods were adapted for non-routine samples including kitchen cloths and spray bottles. Isolates from positive samples were referred to GBRU for WGS and SNP analysis allowing comparison with clinical samples.

## Results

**Figure 1:**
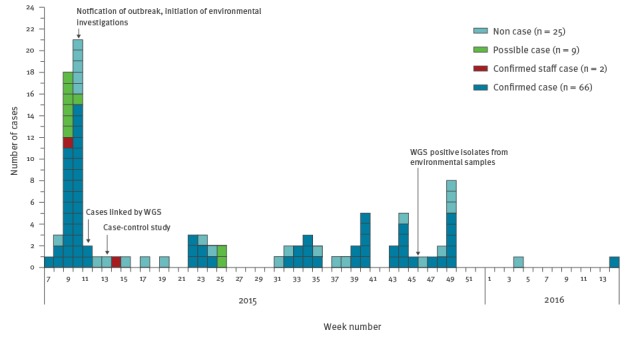
Reported date of symptom onset of cases of a *Salmonella* outbreak and people not meeting case definitions with matching whole genome sequencing of clinical isolates, United Kingdom, Week 7 (February) 2015–Week 14 (March) 2016 (n = 102^a^)

Eighty-two cases (72 confirmed and 10 possible) reported visiting the restaurant. The onset of symptoms (mainly diarrhoea) ranged from 12 February 2015 to 8 March 2016 with apparent point source outbreaks in February and June 2015, followed by a prolonged period of transmission (Figure 1). Four cases (all confirmed) worked in the restaurant kitchen or served food at the restaurant, two of whom were asymptomatic (identified through staff sampling).

WGS: whole genome sequencing.

^a^ Onset dates were not available for two confirmed asymptomatic staff cases, two other confirmed cases, one possible case, and six non cases with matching WGS results (n = 11).

Cases had a median age of 28 years (range: 5 months–85 years), 65% (53/82) were female and 99% (81/82) lived in the same county as the restaurant. Symptoms were mainly self-limiting although 19 cases were hospitalised (1 to intensive care with invasive disease). We also identified 31 people during case finding whose isolates matched the nationally unique WGS outbreak clade but reported not visiting the restaurant, and had no apparent link to cases or takeaway food (n = 13) or declined interview (n = 18). These people did not meet the case definitions, but otherwise did not appear different from cases; median age: 28 years (range: 10 months – 85 years), 52% (16/31) female, with similar symptoms and onset dates, however 19% (6/31) lived in other areas of England or Northern Ireland.

### Case–control study

At the time of the case–control study, 44 cases reported having eaten at the restaurant. Twenty (17 confirmed, 3 possible) responded to the case–control questionnaire (response rate: 20/44). Cases only nominated 10 controls; failing to achieve a 1:1 ratio.

A range of foods, including uncooked salad items were investigated in the case–control study (Table 1). Eating food from the carvery was the only significant (p < 0.01) harmful exposure identified by univariate analysis (OR: 21.37; 95% confidence interval (CI): 2.52 – ∞). All cases had consumed carvery food. Multivariable analysis identified sex and recent antibiotic use, as confounders. The adjusted odds of becoming a case remained over 20 times greater after eating any carvery food (aOR: 20.9; 95% CI: 2.2 – ∞, Table 2). Effect modification was not identified between covariates in the final model. These results were confirmed by sensitivity analyses (data not shown). Throughout the investigation, 94% (60/64) of cases with a known food history reported eating food from the carvery; no cases reported eating salads.

**Table 1:** Results of univariate analysis of food items eaten at a restaurant, implicated in a *Salmonella* outbreak, United Kingdom, March 2015 (n = 30 persons investigated)

Food eaten	Cases			Controls			Crude OR (95% CI)	Exact p value
	Total	Exposed	%	Total	Exposed	%		
**Carvery**	20	20	100	10	5	50	Ind^a^ (4.21–Ind)	0.002
**Any sharing platter**	19	0	0	10	3	30	0 (0.00–0.56)	0.05
**Side dish**	20	0	0	10	2	20	0 (0.00–0.89)	0.1
**Any burger**	20	0	0	10	1	10	0 (0.00–Ind)	0.3
**Any pie**	20	0	0	10	1	10	0 (0.00–Ind)	0.3
**Any fish**	19	0	0	10	1	10	0 (0.00–Ind)	0.3
**Any starter**	19	0	0	10	1	10	0 (0.00–Ind)	0.3
**Any pudding**	20	3	15	10	3	30	0.41 (0.04–3.97)	0.3
**Any drink**	20	18	90	10	9	90	1 (0.02–21.72)	1

CI: confidence interval; ind: indeterminate; OR: odds ratio.

No cases or controls ate items from the following menu sections: jacket potatoes, light bites, salads, steaks, hero dishes, classic dishes.

^a^ Exact logistic regression, median unbiased estimate univariate OR: 21.37 (95% CI: 2.52– ∞).

**Table 2:** Final exact logistic regression multivariable model of carvery food items eaten at a restaurant implicated in a *Salmonella* outbreak, United Kingdom, March 2015 (n = 29^a^)

Exposure	AOR (95% confidence interval)	P value
**Carvery**	**20.9 (2.2–∞)**	**<** **0.007**
**Sex (male)**	2.7 (0.2–148.6)	> 0.7
**Recent antibiotic use**	1.0 (0.7–63.6)	> 0.99

AOR: adjusted odds ratio.

^a^ One case had no record of antibiotic use and was excluded from the model (n = 29), model p < 0.005.

### Clinical microbiological investigations

*S*. Typhimurium was isolated in 75 case samples (72 confirmed, 3 possible); stool samples were not available for seven cases. Phage-type DT193 was identified for 16 isolates, 58 isolates were untypable, and one isolate was not phage-typed. A single MLVA profile was identified (3–14–9-0–0211) in 34 cases (one additional case had a single locus variant). This profile had been associated with human cases and pigs in several regions of England (data not shown). In addition WGS was available for 96% (72/75) of isolates with the first results available 7 days after the start of the investigation, providing increased discrimination over MLVA results.

**Figure 2:**
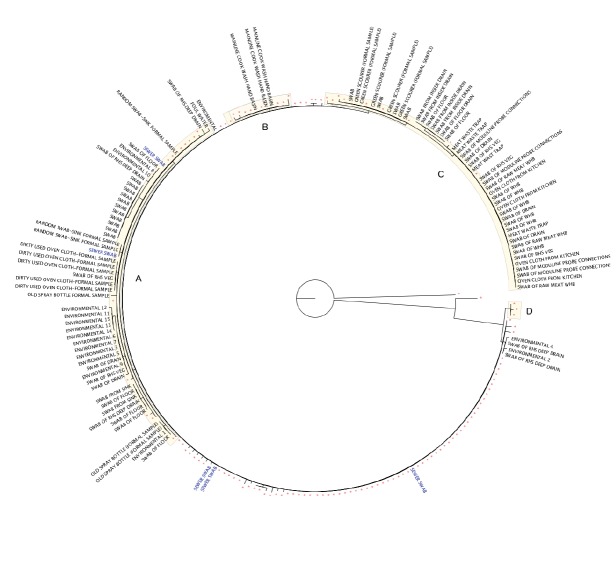
Maximum likelihood phylogenetic tree of clinical (red), sewer (blue) and other environmental samples matching the SNP address 1.1.1.124.395.395 of a *Salmonella* outbreak strain, United Kingdom, February 2015–March 2016 (n = 220 outbreak sequences^a^)

All the isolates clustered within a nationally unique five SNP cluster (which is further referred to as the outbreak clade), 71% (53/72) of isolates were identical across their core genome with no SNP differences observed (outbreak profile). The remaining 19 case isolates varied from the most frequent genotype by between one and five SNPs. An overview of the sequences associated with this nationally unique outbreak profile is shown in the dendrogram (Figure 2) with sequences from environmental sampling, and the additional 31 sequences identified from people during case finding who did not meet case definitions (i.e. non cases). Eight of 13 sequences from non-cases that reported not visiting the restaurant had no SNP difference from the outbreak sequence. Three sub-lineages (Figure 2, marked A–C) which emerged from November 2015 were apparent. Prior to this, genetic variation had been observed, but distinct sub-lineages had not been identified, with the exception of three cases in March 2015 which appeared distinct from the main outbreak lineage (Figure 2, marked D).

SNP: single nt polymorphism.

^a ^114 sequences from clinical isolates (cases and non-cases), this includes sequences where there were multiple samples from cases. 106 sequences from environmental isolates are shown.

An asterisk (*) denotes the position of a clinical isolate in the tree. The first two asterisks at the base of the tree denote the root of the tree and nearest neighbouring sequence.

### Environmental investigations

All routine environmental inspections at the restaurant had been satisfactory before the outbreak. Eighteen inspections were carried out during the investigation; until November 2015 these were inconclusive and therefore the restaurant remained open. There were no reports of staff illness before the outbreak. Carvery food was not available for sampling due to the high food turnover, however, raw meat and fresh water samples were taken at the beginning of each cluster and in early 2016 (n = 21, all negative). The food trace-back investigations were inconclusive and did not identify any suppliers linked with other cases or outbreaks.

Environmental sampling in the kitchen, restaurant, staff rooms, toilets and flats above the restaurant was conducted throughout the investigation, with 354 samples collected (Table 3). These were negative throughout the early stages of the investigation, but from November 2015 onwards *S.* Typhimurium matching the outbreak clade was isolated from 106 environmental samples (Table 3). Isolates from three sewer swabs matched the outbreak profile. From November 2015 samples taken from cleaning materials, the pot wash area and wash hand basins were also found to have isolates that matched the outbreak profile. From January 2016 further areas of the kitchen were implicated along with drains (Table 3). The majority of environmental isolates fell into sub-lineages A–C, however several deep drain swabs were identical to the outbreak sequence (Figure 2).

**Table 3:** Summary of environmental samples (n = 375) with isolates implicated in a *Salmonella* outbreak (n = 106) by whole genome sequencing, United Kingdom, March 2015–May 2016

Sample date	Water samples (n)	Food samples (n)	Environmental samples (n)	WGS positive isolates within five SNP clade(n)	Positive sample source
**Mar 2015**	1	0	26	0	NA
**Jun 2015**	0	1	16	0	NA
**Sep 2015**	1	2	27	0	NA
**Nov 2015**	0	0	19	3	Sinks/wash hand basins
				7	Cleaning materials
				3^a^	Sewer swabs
**Dec 2015**	0	0	67	5	Kitchen cloths
				2^b^	Sinks/wash hand basins
				2^b^	Cleaning materials
**Jan 2016**	6	9	47	15	Surface and deep drain swabs
				4	Kitchen cloths
				12^a^	Sinks/wash hand basins
				4	Meat sink waste trap
				7	Floor swabs
				10	Kitchen surface swabs
**Feb 2016**	1	0	42	19	Surface and deep drain swabs
				1	Sinks/wash hand basins
**Mar 2016**	0	0	41	12	Surface and deep drain swabs
**Apr 2016**	0	0	59	0	NA
**May 2016**	0	0	10	0	NA
**Total**	9	12	354	106	NA

NA: not applicable; SNP: single nucleotide polymorphism; WGS: whole genome sequencing.

^a^ Three isolates matched outbreak sequence (0 SNP difference).

^b^ One isolate matched outbreak sequence (0 SNP difference).

Mapping and visual inspection of the drainage systems identified significant issues. Water filled traps (u-bends) designed to prevent foul air flow from the drainage system into the building had failed and smoke testing revealed some ineffective drain seals, potentially allowing contaminated bio-aerosol to be disseminated into the kitchen. One sink drain was not connected to any drainage system with waste water pooling under the floor. Other larger drains had failed after leaking waste-water washed away the supporting substrate forming a cavity under the kitchen area. It transpired at that point that drainage water had, on occasion, risen into the kitchen area, although this had not been previously reported. Substantial remedial works were undertaken, however, these were found to have failed on re-inspection and so these drains were later decommissioned.

### Interventions

The restaurant responded to the outbreak by rotating staff away from carvery duties in case they were unsuspecting carriers, retraining staff on hygiene procedures, facilitating voluntary staff sampling, installing and monitoring additional hand washbasins, moving raw meat storage outside of the kitchen, and conducting deep cleans of the indoor environment, which over the outbreak increased in frequency and progressed to include weekly steam cleaning and hydrogen peroxide fogging. Cameras were also installed to monitor the carvery buffet, following concerns that deliberate contamination may have occurred.

The restaurant voluntarily closed for 2 days for cleaning following positive environmental swab results in November 2015 and again for several weeks after further positive sampling to allow deep cleaning, kitchen refitting, and restaurant refurbishment. Remedial work was undertaken on the drains in February 2016. Capping of all kitchen floor drains and hydrogen peroxide fogging followed in March 2016. This appeared to resolve issues of contamination. Throughout the investigation EHOs and PHE staff remained in close contact with the restaurant and provided advice.

## Discussion

Our investigation identified a prolonged outbreak of *S*. Typhimurium linked to a restaurant where 82 cases (72 confirmed, 10 possible) had eaten food. There was strong epidemiological evidence of a link to the restaurant early in the investigation, supported by timely WGS results which provided a highly discriminatory microbiological link between cases and confirmed an initial point source outbreak. In total, 72 case isolates and 106 environmental isolates from the restaurant clustered into a unique 5 SNP cluster with 61 isolates (53 from cases and eight from environmental samples) identical at the core genome level. However, the environmental link with the restaurant was not established until 7 months after the start of the outbreak when WGS positive isolates were obtained from the kitchen and drainage system.

We found the drains had failed in several places and hypothesised that a reservoir of bacteria in biofilm [15] and flooded areas in underfloor cavities may have sustained this outbreak, after repeated environmental cleaning failed. Drainage problems in one area of the kitchen led to liquid from the drains seeping into the kitchen suggesting a contamination pathway. We found isolates matching the outbreak strain on kitchen cloths, swabs from kitchen sinks, and pot wash areas suggesting contact with sinks may have provided a second contamination pathway. We also identified ineffective drain water-traps potentially allowing the movement of contaminated bio-aerosols [13]. Smoke tests demonstrated the potential for dissemination of foul air into the kitchen.

While aerosolised drain contamination has not been previously described in *S*. Typhimurium outbreaks, there is supporting evidence for this transmission pathway for Gram-negative bacteria in hospital drainage systems (modelled using *Pseudomonas putida*) [13]. Bio-aerosol transmission in building drains was implicated in a large outbreak of severe acute respiratory syndrome suggesting this as a feasible hypothesis [16]. Previous outbreak investigations have also identified the prolonged survival of food-borne pathogens such as *Listeria monocytogenes* in drainage systems of food production plants [15,17]. Sinks and drains have also been linked with prolonged bacterial outbreaks in hospital settings [18,19]. Occult environmental contamination can therefore be difficult to identify and control in diverse settings.

There was strong epidemiological evidence that eating carvery food was associated with illness, suggesting it may have been an important transmission vehicle. However, there was no other evidence to confirm contamination of food or utensils, and temperature logs suggested food was cooked appropriately therefore the cooked food must have been contaminated in the kitchen environment if this hypothesis is correct. Direct transmission from asymptomatic staff could also not be ruled out as not all staff complied with the first round of screening. We could not rule out the effect of potential lapses in cleaning and kitchen hygiene practices that may also have sustained this outbreak. Eating at busy weekend periods appeared to be linked to cases in the descriptive analysis, suggesting working practices may have been less rigorously applied during busy periods, even after staff received additional hygiene training during the outbreak.

Many of the isolates sampled from drains belonged to one of several genetic sub-lineages which had evolved from the main outbreak lineage. Several *Salmonella* isolates sampled from the drains also matched the main outbreak sequence supporting the hypothesis that the drains were potentially an important reservoir. The kitchen drains and sewer system were unlikely to be the original source and may have become contaminated by asymptomatic staff, or a diner [20]. It is also possible that a one-off contamination event from raw or undercooked food was responsible for seeding this outbreak as raw meats such as pork cannot be guaranteed to be *Salmonella* free, although our food trace-back did not find a supply chain problem.

We were unable to identify sources of infection or transmission routes for the 31 people infected with the outbreak strain (13 reported no restaurant exposure). We considered that some of these people may have been unknowing secondary cases, given the national unique WGS results, which had not been observed before or since this outbreak, and also that asymptomatic carriage was observed during the outbreak, however no epidemiological links to outbreak cases with restaurant exposure were identified.

### Strengths and limitations

A key strength of this investigation was the use of routine WGS which strengthened the epidemiological evidence of a point-source outbreak enabling rapid implementation of control measures before a holiday weekend in March 2015. However, there were also a number of limitations. Early cases noted their suspicions of the restaurant on social media which may have introduced recall bias in our case–control study, inflating measures of association. The analytical study had a poor response rate and a small sample size. This possibly introduced type II error for other food exposures or potential confounders which may partially explain why other foods were not identified as potential sources of infection even though contamination in the kitchen was possible. Our analysis plan excluded protective food exposures potentially increasing residual confounding in our final model.

Other information biases may have been present in this investigation as many cases were identified after the hypothesis had been established, potentially leading to over ascertainment of exposure at the restaurant. However, the investigation did not identify a common source other than the restaurant, no other outbreaks with the unique profile have been identified and since the drainage issues have been resolved, no further cases have been identified.

Evidence of environmental contamination was lacking before November 2015. This was possibly due to the lower density and frequency of sampling or the techniques used, although it is unclear if these or other factors resulted in inconclusive findings.

### Implications for public health

*Salmonella* outbreaks are commonly linked to food, restaurants and water supplies [21-26]. Biofilm may harbour *Salmonella* sp. in drains and long-term environmental contamination is possible [3], but rarely reported nor is the potential for bio-aerosol related contamination. Our findings suggest greater consideration should be given to undertaking drain swabbing at an early stage of restaurant and food related outbreak investigations. This will enable identification of similarities between environmental and clinical isolates that may have previously not been possible before the routine use of highly discriminatory WGS.

This outbreak was unexpected in a newly built restaurant managed by a national chain. Defective drains may have been a one-off incident. However, repeated failures of drains and continued contamination of the kitchen identified here suggests the design and/or installation of drainage systems was suboptimal. Increased inspections in the building process may be required. Consideration should be given to drainage systems which facilitate inspection of water-traps or use of multiple traps should be considered to reduce the impact of failure.

### Conclusions

This outbreak of a nationally unique strain of *S.* Typhimurium was linked to a single restaurant. The defective drainage system in the restaurant may have acted as an environmental reservoir and dissemination of bio-aerosol from the drains into the kitchen was plausible. This linked with possible lapses in kitchen hygiene may have enabled intermittent contamination of food during the period of the outbreak. While the original source remains unclear, this protracted outbreak was controlled after remedial work on the drains, a kitchen and restaurant refurbishment and a deep clean involving hydrogen peroxide fogging.

Public health professionals should consider drainage systems and bio-aerosols as potential sources in any outbreak of salmonellosis and environmental investigations should include swabbing drains early in outbreak scenarios. Investigators should work towards accessing timely WGS analysis, which in this investigation was essential for case finding, establishing a single most probable source and underpinning the epidemiological evidence used to demonstrate a need for actions to prevent further cases.
